# Cilengitide, an αvβ3-integrin inhibitor, enhances the efficacy of anti-programmed cell death-1 therapy in a murine melanoma model

**DOI:** 10.1080/21655979.2022.2029236

**Published:** 2022-02-10

**Authors:** Xin Pan, Minxiao Yi, Chaofan Liu, Yu Jin, Bo Liu, Guangyuan Hu, Xianglin Yuan

**Affiliations:** Department of Oncology, Tongji Hospital, Tongji Medical College, Huazhong University of Science and Technology, Wuhan, Hubei Province, China

**Keywords:** Melanoma, immune checkpoint inhibitor, αvβ3-integrin, cilengitide, PD-1 blockade

## Abstract

Integrins play an important role in multiple stages of tumor progression and metastasis. Previous studies have shown synergistic effects of combined αvβ6-integrin and αvβ8-integrin inhibitors with immunotherapy. However, the role of αvβ3-integrin inhibitor in tumor immunity is still unclear. In this study, we aimed to elucidate the impact of the αvβ3-integrin inhibitor on PD-L1 expression and sensitivity to immune checkpoint blockade in melanoma. We investigated the effects of cilengitide, an αvβ3-integrin inhibitor, on cell viability and apoptosis of melanoma cell lines. And we explored how cilengitide regulated the expression of PD-L1 in melanoma cells in vitro and in vivo, using immunofluorescence, flow cytometry, Western blotting, and immunohistochemistry. A subcutaneous B16 murine melanoma model was utilized to determine whether combining cilengitide with anti-PD1 therapy inhibited tumor growth and positively regulated tumor microenvironment (TME). Our results showed that cilengitide inhibited cell viability and induced apoptosis in B16 and A375 cell lines. Furthermore, cilengitide decreased PD-L1 expression by reducing STAT3 phosphorylation in two melanoma cell lines. Cilengitide also reduced subcutaneous tumor PD-L1 expression in the B16 murine melanoma model. Accordingly, cilengitide positively regulated antitumor immune responses and provided durable therapy when combined with anti-PD1 monoclonal antibody in the murine melanoma model. This combination therapy reduced tumor growth and extended survival. Our study highlights that cilengitide enhances the efficacy of anti-PD1 therapy and produces a stronger antitumor immune response. This combination therefore represents a novel therapeutic regimen that may improve immunotherapy treratment.

## GRAPHICAL ABSTRACT

**Figure:**
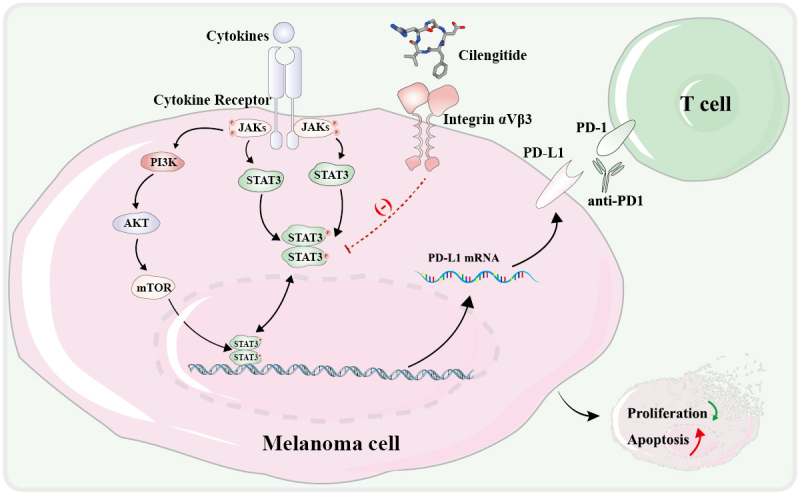

## Introduction

Melanoma is one of the leading causes of cancer mortality worldwide [1]. It has a poor prognosis owing to rapid metastasis and malignancy [2]. For decades, many attempts have been made to develop satisfactory treatments for melanoma patients [3–5]. In recent years, melanoma treatments have been extended with the evolution of immunotherapies, especially immune checkpoint inhibitors [6,7]. Programmed cell death-1 (PD-1)/programmed cell death-ligand 1 (PD-L1) blockade has elicited substantial responses in advanced and metastatic malignant melanoma [8,9]. Unfortunately, only a part of patients responds to single-agent immunotherapy. 40 to 65% of melanoma patients show little or no response to immune checkpoint blockade [10,11]. Understanding the mechanisms that determine response and resistance to therapy is critical to improving outcomes. Combining immunotherapy such as PD-1/PD-L1 blockade with other treatments is expected to improve the efficacy and broaden the range of individuals who may benefit [12,13].

Integrins are multifunctional heterodimeric transmembrane receptors expressed on several cell types that regulate a variety of crucial functions, including cell survival, proliferation, migration, and neoangiogenesis [14–16]. It has been proved that blocking of integrin function can effectively inhibit the initiation, progression, and metastasis of various cancer types [17–19]. Cilengitide is a high-affinity αvβ3-integrin antagonist that was first developed as an antiangiogenic drug to treat glioblastoma and other cancers [20,21]. However, the phase III clinical trial of cilengitide in glioblastoma did not achieve satisfactory endpoints, the efficacy of cilengitide has thus come under question [20]. Recent studies have focused on the role of cilengitide in fibrotic disease, since cilengitide inhibits transforming growth factor β (TGF-β)/Smad signaling [22,23].

Previous studies have shown that the elevated expression of αvβ8-integrin and αvβ6-integrin on the surface of tumor cells caused tumor immune escape [24,25]. However, the functional role of αvβ3-integrin in cancer immunotherapy is not well understood and needs to be further studied. Here, we aimed to investigate the potential of using an αvβ3-integrin inhibitor in combination with anti-PD1 monoclonal antibody to improve the treatment response in melanoma. In this study, we explored the role of cilengitide on PD-L1 expression in melanoma cell lines and in a murine melanoma model. Besides, we evaluated the therapeutic effects of combined cilengitide and anti-PD1 antibody in a murine melanoma model, including characterizing CD4^+^ and CD8^+^ tumor infiltrating lymphocytes (TILs) in both subcutaneous tumors and spleens of mice.

## Materials and Methods

### Cell lines and reagments

B16 cells were purchased from the China Center for Type Culture Collection (CCTCC, Wuhan, China). The certificate of cell purchase was presented in Supplementary Fig. 1. A375 and HaCat cells were the generous gifts from the Laboratory of Dermatology, Tongji Hospital (Wuhan, China). All cells were cultured in dulbecco’s modified eagle medium (DMEM, Hyclone, Provo, UT, USA) contained with 10% fetal bovine serum (FBS, Gibco, Rockville, MD, USA) at 37°C in a 5% CO_2_ incubator.

### Study drugs

Anti-PD1 monoclonal antibody (clone: RMP1-14) and an isotype control were purchased from BioXCell (West Lebanon, NH, USA) and stored at 4°C. Cilengitide was purchased from MedChemExpress (Monmouth Junction, NJ, USA) and dissolved in dimethyl sulfoxide (DMSO) and stored at −20°C. Recombinant mouse interleukin-6 (IL-6, HY-P7063) and recombinant human IL-6 (HY-P7044) were purchased from MedChemExpress, dissolved in phosphate buffered saline (PBS, Promotor, Wuhan, Hubei, China) and stored at −20°C.

### Cell viability assays

Cells (6 × 10^3^ per well) were seeded in 96-well plates and incubated overnight. Cells were then treated with cilengitide at a concentration of 0, 1, 10, 100, and 1000 µg/ml. After 24 hours, 48 hours, and 72 hours, 10 µL of the Cell Counting Kit (CCK)-8 solution (MedChemExpress) was added to each well. After incubation for 2 hours at 37°C and 5% CO_2_, the absorbances were measured at 450 nm in a microplate reader (BioTek, Winooski, VT, USA).

### Colony formation assays

For the colony formation experiment, cells (5 × 10^2^ per well) were seeded in 6-well plates in the presence or absence of 5 µg/ml and 10 µg/ml cilengitide and cultured at 37°C for two weeks. Cells colonies were stained with 0.1% crystal violet for 15 minutes before being counted under a microscope. Colonies containing more than 50 cells were counted.

### Immunofluorescent staining of cells

Cells (1 × 10^4^ per well) were seeded into 24-well plates and incubated in the presence or absence of 5 µg/ml cilengitide. After 12 hours, cells were fixed with paraformaldehyde for 15 minutes and blocked with 5% bovine serum albumin (BSA, Promotor) in phosphate buffered saline tween (PBST, Promotor) solution for 1 hour. Then, 200 µl PD-L1 antibody (1:100, Proteintech, Rosemont, IL, USA) was added to each plate and incubated at 4°C overnight, followed by the Alexa Fluor 488 secondary antibody (1:100, Promotor) at room temperature in dark for 1 hour. Staining with 4’,6-diamidino-2-phenylindole (DAPI, Promotor) was carried out for 5 minutes, and visualized under a fluorescence microscope.

### Western blot analysis

Protein was extracted from cells using radioimmunoprecipitation assay (RIPA) lysis buffer (Beyotime, Shanghai, China), phenylmethylsulfonyl fluoride (PMSF), and phosphatase inhibitors (Promotor). After 10% sodium dodecylsulfate polyacrylamide gel electrophoresis (SDS-PAGE), the extracted protein was transferred to a 0.45 µm polyvinylidene fluoride (PVDF) membrane (Millipore, Bedford, MA, USA). Then, the membrane was incubated with the indicated primary antibodies (1:1000) at 4°C overnight. Details of the antibodies were presented in Supplementary Table 1. Then, the membrane was incubated with secondary antibodies (1:5000, Promotor) at room temperature for 1 hour, and visualized using SuperSignal West Pico Chemiluminescent Substrate (Thermo Scientific, Waltham, MA, USA).

### Real-time quantitative polymerase chain reaction (RT-qPCR)

Total RNA was extracted from cells using TRIzol reagent (Beyotime, Shanghai, China) and cDNA was obtained by reverse transcription using Hiscript II Q RT supermix (Vazyme Biotech co.,ltd, Nanjing, Jiangsu, China). RT-qPCR was completed on a 7900 HT fast real-time PCR system using SYBR qPCR master mix (Vazyme Biotech co.,ltd). The primer sequences used were listed in Supplementary Table 3. The data were analyzed by 2^−ΔΔCt^ method, and GAPDH was used as the reference.

### RNA sequencing

B16 cells were cultured in the presence or absence of 5 µg/ml cilengitide for 12 hours. Total RNA was extracted using TRIzol reagent (Beyotime), and next generation sequencing library preparations were constructed according to the manufacturer’s protocol (Thermo Scientific, Waltham, MA, USA). The libraries were sequenced on Illumina HiSeq instrument (Illumina, San Diego, CA, USA). Sequences were processed and analyzed by GEWIZ. Adjusted p-value < 0.05 and fold change > 2 were set as the criterion for significant differential expression. The original data has been uploaded to the NCBI database (PRJNA728363).

### Allograft tumor model, immunohistochemistry (IHC), and immunofluorescent staining of tumors

Female C57BL/6 mice aged 6–8 weeks were obtained from the Hunan SJA Laboratory Animal Co., Ltd. (Changsha, Hunan, China), and raised in a specific pathogen-free (SPF) animal laboratory. All experiments were approved by the institutional animal care and use committee at Tongji Medical College (IACUC Number: 2383). B16 cells (5 × 10^5^ cells in 200 μl PBS) were injected subcutaneously into the right side of the mice. Eight days after tumor implantation, when tumors reached approximately 100 mm^3^, mice were randomly divided into four groups (n =  6 per group). Cilengitide (50 mg/kg) or PBS was administered intraperitoneally daily. The treatment was determined according to the guide for dose conversion between animals and humans, as well as animal experimental methods from previously published studies [20,26]. Anti-PD1 monoclonal antibody (10 mg/kg) or isotype control (10 mg/kg) was administered intraperitoneally every three days. The tumor volume was measured every two days and calculated as follows: [(length) × (width^2^)]/2. The weight of mice was measured every three days. The endpoint of observation was defined as either mouse death or tumor volume reached 2000 mm^3^. Mice were euthanized at 21 days. Tumors were collected, weighed, and soaked in paraformaldehyde. Tumor tissues were embedded into paraffin blocks and stained according to the IHC kit protocol (Abcam, Cambridge, MA, USA). Multicolor immunofluorescence staining was implemented on the basis of the manufacturer’s guidelines of immunofluorescence staining kit (Beyotime).

### Enzyme-linked immunosorbent assay (ELISA)

Blood and tumor tissues from the mice were collected for ELISA detection. The blood samples were placed at room temperature for 2 hours and centrifuged at 1000 xg for 20 minutes to obtain serum. Tumor tissues were cut into small pieces and ground on ice to collect tissue homogenates. Interferon-γ (IFN-γ) and granzyme B levels in serum and tumor tissues were determined according to the operational guidance of ELISA kits (1132, 1660, ELK Biotechnology, Wuhan, Hubei, China).

### Flow cytometry

For the apoptosis assay, cells (5 × 10^5^ per well) were seeded into 6-well plates and incubated in the presence or absence of 5 µg/ml and 10 µg/ml cilengitide for 12 hours. Cells were stained with FITC Annexin V and propidium iodide (PI) according to the manufacturer’s guidelines of apoptosis detection kit (BD, Franklin Lakes, NJ, USA). Cells were measured immediately by flow cytometry (Calibur, BD Bioscience, Franklin Lakes, NJ, USA). For the evaluation of PD-L1 expression on the cell surface, cells (5 × 10^5^ per well) were seeded into 6-well plates and incubated in the presence or absence of 5 µg/ml cilengitide for 12 hours. Cells were digested and incubated with 0.3 µl PD-L1 antibody (1:100, Biolegend, San Diego, CA, USA) in 30 µl of PBS for 30 minutes at 4°C. The proportion of PD-L1 positive cells was detected using flow cytometry (Calibur). For the analysis of tumor and spleen tissues, we isolated tumor tissues and removed blood and necrosis. Tumor tissues were immersed in the prepared digestive reagent containing 1 mg/ml collagenase IV, 0.01% hyaluronidase, and 0.002% DNase I (Promotor) in a 37°C water bath for 30 minutes. Spleens were gently ground in PBS containing 0.1% FBS. Cells were centrifuged at 1200 rpm for 5 minutes, then the supernatant was discarded. The live cells were collected, counted, and resuspended in PBS containing 0.1% FBS. Subsequently, we added fluorescent antibodies (1:100, Biolegend, San Diego, CA, USA) specific to mouse CD45, CD3ε, CD4, and CD8α to the tumor cell suspensions, and fluorescent antibodies specific to mouse CD3ε, CD4, and CD8α to the spleen cell suspensions. Details of the antibodies are presented in Supplementary Table 2. Tumor and spleen suspensions were all incubated for 30 minutes at 4°C. Antibodies were removed and cells were resuspended in PBS, before analysis using a flow cytometer (CytoFLEX LX, Beckman Coulter, Krefeld, Germany). Data were analyzed using FlowJo software (Version 10.0, San Carlos, CA, USA).

### Bioluminescent imaging of tumors

A lentiviral vector expressing the firefly luciferase gene was constructed (Tsingke Biotechnology Co., Ltd, Beijing, China) and transfected into B16 cell lines. B16 cell lines stably expressing the firefly luciferase gene (B16-luc) were obtained by purinomycin screening. B16-luc cells (5 × 10^5^ cells in 200 μl PBS) were injected subcutaneously into the right side of C57BL/6 mice. Mice were anesthetized using 1% pentobarbital sodium and intraperitoneally injected with 1.5% D-luciferin. Images were acquired using the Xenogen IVIS Spectrum system (Caliper) on days 8 and 20 after tumor implantation.

### Statistical analysis

Statistical analysis and interpretation were conducted in GraphPad Prism v8 software (La Jolla, CA, USA). Survival curves were plotted using Kaplan-Meier statistical analysis, and the log-rank test was used to determine the statistical significance. Statistical comparisons between two groups were analyzed using unpaired Student’s t-tests. Data were presented as mean ± standard deviation. P value < 0.05 (*) was considered statistically significant. Each experiment was repeated three times.

## Results

The purpose of this study was to investigate the effects of αvβ3-integrin inhibitor on the proliferation and apoptosis of melanoma cells, and the sensitivity of immune checkpoint inhibitors. Our results revealed that cilengitide inhibited proliferation and increased apoptosis in melanoma cells in vitro. Addtionally, cilengitide downregulated the expression of PD-L1 on melanoma cells by reducing STAT3 phosphorylation. Besides, cilengitide enhances the function of CD8^+^ T cells when combined with anti-PD1 monoclonal antibody in B16 murine melanoma model, providing a promising therapeutic method for improving the response rate to immunotherapy.

### β3-integrin is overexpressed in human skin cutaneous melanoma

**Figure 1.**
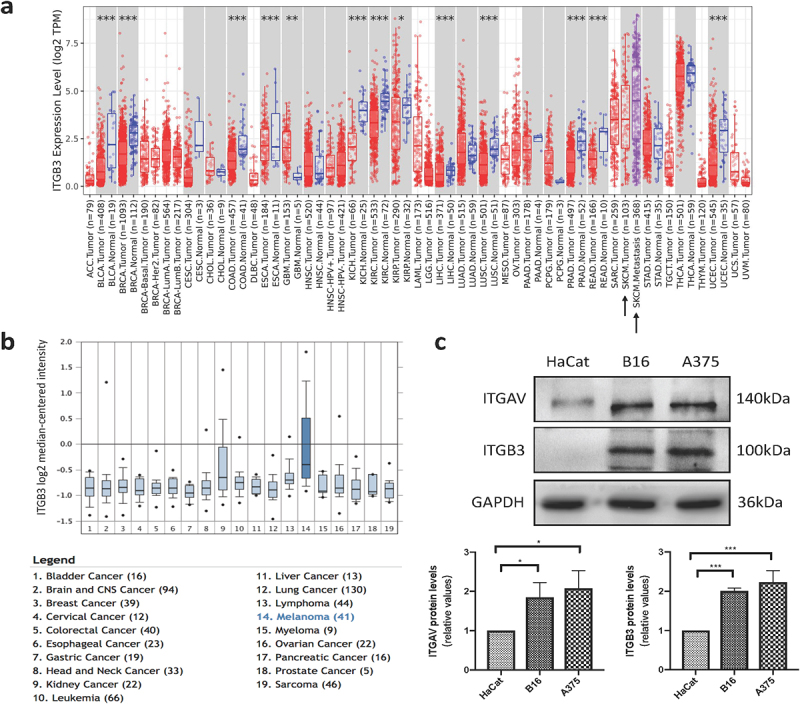
**β3-integrin is highly expressed in melanoma**. (a) The expression level of β3-integrin in different tumor types in TIMER database. (b) The expression level of β3-integrin in different tumor types in Oncomine database. (c) The expressions of αv-integrin and β3-integrin in HaCat, B16, and A375 cells, detected by Western blotting.

To investigate the differences in β3-integrin expression levels between multiple types of tumor tissue and normal tissue, we first used online databases including Oncomine and Tumor Immune Estimation Resource (TIMER). We analyzed the expression levels of β3-integrin in various tumor types using the TIMER database. As shown in Figure 1(a), the expression levels of β3-integrin were significantly higher in skin cutaneous melanoma (SKCM) and metastatic skin cutaneous melanoma (SKCM-metastasis). Then, we reconfirmed the transcriptional expression of β3-integrin in different tumor types using the Oncomine database. The results revealed that the expression of β3-integrin was markedly higher in SKCM samples than in other types of tumors (Figure 1(b)). We further examined the protein expression of β3-integrin in melanoma and normal skin tissue in the Human Protein Atlas (HPA). As shown in Supplementary Fig. 4a, β3-integrin was overexpressed in melanoma compared with normal skin tissue. In general, β3-integrin was highly expressed in melanoma, indicating that it may be a promising target for the treatment of melanoma. We then evaluated the expression of αv-integrin and β3-integrin in the two melanoma cell lines (B16 and A375) and human keratinocyte line (HaCat). Western blotting results showed that αv-integrin and β3-integrin expressed in both B16 and A375 cells. Moreover, the expression of αv-integrin and β3-integrin in melanoma cells was increased compared with normal skin keratinocyte cells (Figure 1(c)).

### Cilengitide affects genes related to cell growth and apoptosis process in melanoma cells

**Figure 2.**
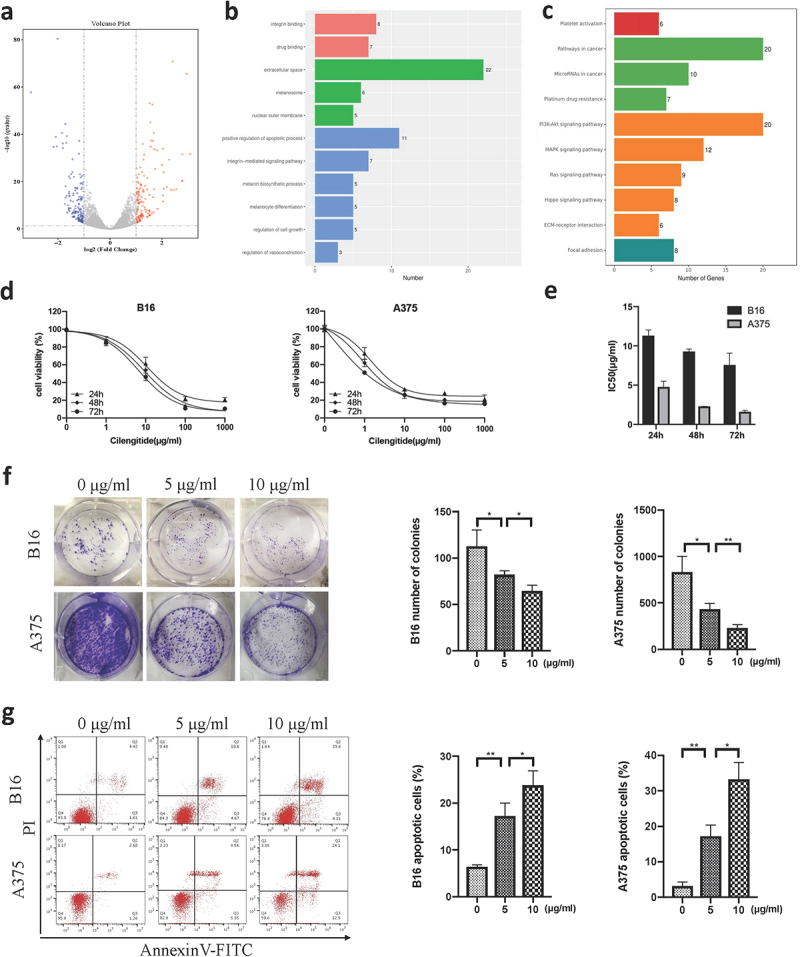
**Cilengitide decreases cell viability and induces apoptosis of B16 and A375 cells in vitro**. (a) RNA sequencing results of normal B16 cells and cilengitide treated B16 cells. (b) Eleven enriched pathways with cilengitide treatment identified by GO analysis. (c) Ten enriched pathways with cilengitide treatment identified using KEGG classification. (d) Viability of B16 and A375 cells treated with cilengitide detected by CCK-8 assay. (e) The IC50 values for cilengitide in B16 and A375 cells. (f) Cell colony numbers of B16 and A375 cells treated with 0, 5, and 10 µg/ml cilengitide. (g) Flow cytometry was used to detect B16 and A375 cells apoptosis after treatment with 5 µg/ml and 10 µg/ml cilengitide. Data were represented as mean ± standard deviation. *p < 0.05, **p < 0.01, ***p < 0.001, NS, not significance (Student’s t test). Each experiment was repeated three times.

To better understand the effects of cilengitide on melanoma cells, we sequenced the transcriptome of untreated B16 cells and B16 cells treated with 5 µg/ml cilengitide for 12 hours. Adjusted p-value < 0.05 and fold change > 2 were set as the thresholds for screening differentially expressed genes (DEGs). A total of 303 genes were altered, 140 of which were upregulated and 163 were downregulated (Figure 2(a)). Gene Ontology (GO) analysis showed changed terms in cilengitide treated B16 cells compared with untreated B16 cells. Changes of integrin binding, integrin-mediated signaling pathway, extracellular space, and nuclear outer membrane indicated that cilengitide affected cell morphology and adhesion. Meanwhile, cilengitide had an impact on cell growth and apoptotic process. Interestingly, the results reflected the enrichment of melanosome, melanin biosynthetic process and melanocyte differentiation. In addition, the expression of seven genes involved in drug binding and three genes involved in regulation of vasoconstriction were alerted with cilengitide treatment (Figure 2(b)). Kyoto Encyclopedia of Genes and Genomes (KEGG) analysis showed that the two best characterized pathways involved in the regulation of cell growth and apoptosis, the PI3K-AKT and MAPK signaling pathways, were the most enriched (environmental information processing). We verified the expression of the PI3K-AKT and MAPK signaling pathway related genes by RT-qPCR (Supplementary Fig. 4b). Then we detected the phosphorylation of key molecules of PI3K-AKT pathway. We found that the phosphorylation of AKT and mTOR was significantly decreased after treatment with 5 µg/ml cilengitide for 12 hours compared with untreated B16 cells (Supplementary Fig. 4c). Furthermore, the Ras and Hippo signaling pathways were also enriched (Figure 2(c)). According to the sequencing results, we found that cilengitide altered the expression of genes involved in cell growth and apoptosis process in B16 cells, providing a theoretical basis for our subsequent experiments.

### Cilengitide decreases cell viability and induces apoptosis in melanoma cells in vitro

We explored the effect of cilengitide on melanoma cells using several in vitro experiments. First, two melanoma cell lines, B16 and A375, were cultured with a sequence of concentrations of cilengitide (0, 1, 10, 100, and 1000 µg/ml) for 24 hours, 48 hours, and 72 hours. Cilengitide inhibited B16 and A375 cells growth in a time-and dose-dependent manner (Figure 2(d)). The 50% inhibitory concentrations (IC50) of cilengitide on the two cell lines are shown in (Figure 2(e)). To further confirm the inhibitory effect of cilengitide on the proliferation of B16 and A375 cells, we performed a colony formation assay, and fewer colonies were observed after 5 µg/ml and 10 µg/ml cilengitide treatment (figure 2(f)). In addition, the apoptosis rates of B16 and A375 cells in the cilengitide-treated groups were significantly higher than those in the control groups (Figure 2(g)). The apoptosis rates of B16 and A375 cells were 15.27% and 14.89% after incubated with 5 µg/ml cilengitide for 12 hours; 21.71% and 36.6% after incubated with 10 µg/ml cilengitide for 12 hours. In conclusion, our data strongly indicated that cilengitide decreased cell viability and induced apoptosis in melanoma cells in vitro.

### Cilengitide suppresses STAT3 phosphorylation and PD-L1 expression in melanoma cells in vitro

**Figure 3.**
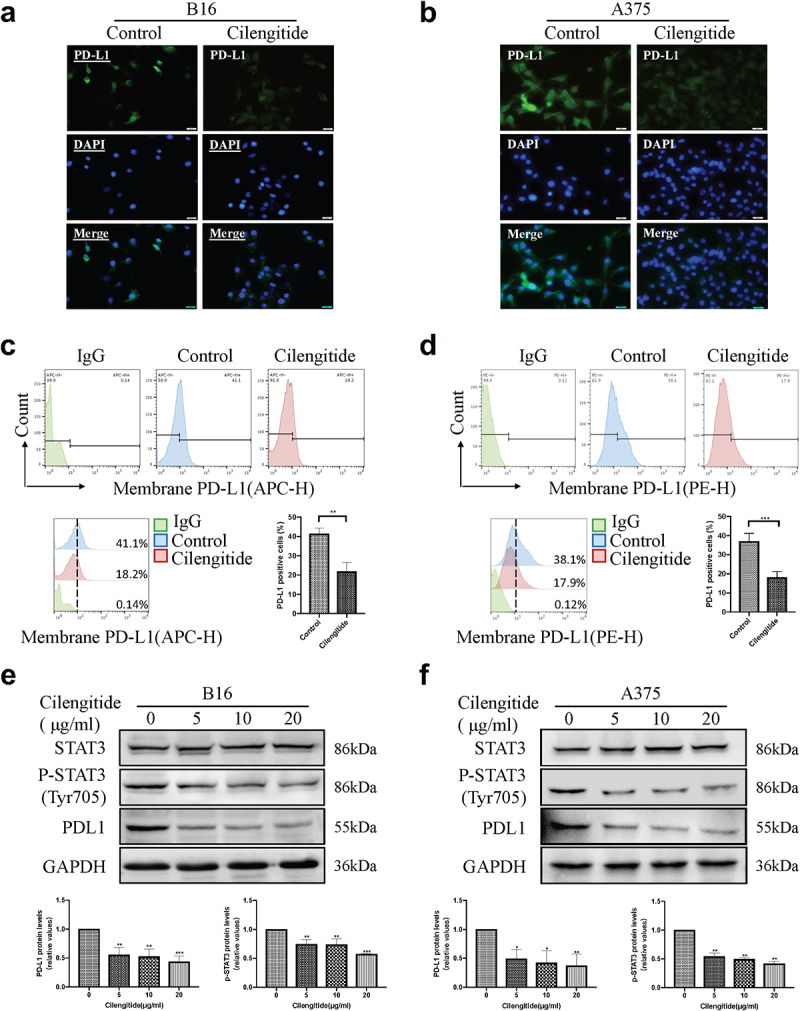
**Cilengitide decreases the expression of PD-L1 in B16 and A375 cells**. (a and b) Fluorescence microscopic images showing the expression of PD-L1 in B16 and A375 cells treated with or without 5 µg/ml cilengitide for 12 hours. Scale bar: 50 µm. (c and d) The proportion of PD-L1 positive B16 and A375 cells detected by flow cytometry after treatment with 5 µg/ml cilengitide for 12 hours. (e and f) The expressions of STAT3, p-STAT3, and PD-L1 detected by Western blotting in B16 and A375 cells after treatment with 0, 5, 10, and 20 µg/ml cilengitide for 12 hours. Data were represented as mean ± standard deviation. *p < 0.05, **p < 0.01, ***p < 0.001, NS, not significance (Student’s t test). Each experiment was repeated three times.

**Figure 4.**
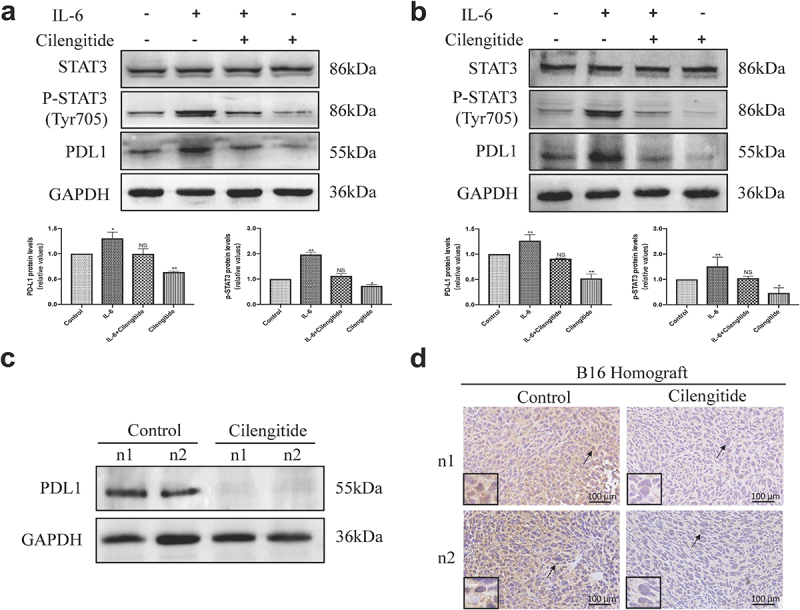
**Cilengitide downregulates the expression of PD-L1 via STAT3 pathway and decreases the expression of PD-L1 in B16 murine melanoma model**. (a and b) B16 and A375 cells were treated with blank control, 20 ng/ml IL-6, 5 µg/ml cilengitide, or 20 ng/ml IL-6 plus 5 µg/ml cilengitide for 12 hours. The expressions of STAT3, p-STAT3, and PD-L1 was detected by Western blotting. (c) B16 cells were inoculated subcutaneously into the C57BL/6 mice, and the mice were treated with 50 mg/kg cilengitide for 7 days. Tumor tissues were then isolated and the expression of PD-L1 was detected by Western blotting. (d) The PD-L1 expression of tumor tissues was detected by immunohistochemical staining. Scale bar: 100 µm. Data were represented as mean ± standard deviation. *p < 0.05, **p < 0.01, ***p < 0.001, NS, not significance (Student’s t test). Western blotting was repeated three times.

Previous studies have shown that the expression of PD-L1 was associated with the efficacy of immune checkpoint inhibitors in melanoma [27,28]. Therefore, understanding the molecular regulation of PD-L1 expression may help to predict the therapeutic potential of cilengitide in combination with immunotherapy. We investigated the effect of cilengitide on the expression of PD-L1 on melanoma cells using three different cell membrane protein detection methods. In an immunofluorescent assay, PD-L1 expression was downregulated in both B16 and A375 cells after treatment with 5 µg/ml cilengitide for 12 hours (Figure 3(a&b)). Flow cytometry analysis was used to confirm the downregulation of PD-L1 expression on B16 and A375 cells caused by cilengitide treatment. The rates of PD-L1 positive expression in the IgG control group, untreated, and 5 µg/ml cilengitide-treated B16 cells were 0.14%, 41.1%, and 18.2% (Figure 3(c)). The corresponding rates in the A375 cells were 0.12%, 38.1%, and 17.9%, respectively (Figure 3(d)). To explore the mechanism of cilengitide-mediated PD-L1 downregulation, we treated B16 and A375 cells with cilengitide at a concentration of 0, 5, 10, and 20 µg/ml and detected both the expression of PD-L1 and the phosphorylation of STAT3 (Tyr705). We found that cilengitide suppressed PD-L1 expression and STAT3 phosphorylation at concentrations greater than 5 µg/ml. There was no significant difference in total STAT3 protein among the four groups (Figure 3(e&f)). The IL-6 family regulates STAT3 activation, and has been used to stimulate STAT3 phosphorylation in previous studies [29–31]. We therefore stimulated B16 and A375 cells with IL-6 to observe whether increased phosphorylation of STAT3 reversed the cilengitide-induced downregulation of PD-L1 expression. STAT3 phosphorylation was significantly increased after incubation with 20 ng/ml IL-6 for 12 hours, and the expression level of PD-L1 was also increased compared with the control group. When B16 and A375 cells were co-treated with IL-6 and cilengitide, the suppression of PD-L1 expression with cilengitide treatment disappeared, with no significant difference in PD-L1 expression between the combined treatment group and the control group (Figure 4(a&b)). These results showed that cilengitide reduced PD-L1 expression by decreasing STAT3 phosphorylation.

### Cilengitide suppresses PD-L1 expression in vivo

To verify the role of cilengitide in regulating PD-L1 expression in vivo, we inoculated B16 cells subcutaneously into the right side of C57BL/6 mice. The mice were then injected intraperitoneally with 50 mg/kg cilengitide for 7 days. After 7 days, tumor tissues were isolated and the expression of PD-L1 was detected by Western blotting. The PD-L1 expression was reduced in tumors from mice treated with cilengitide compared with untreated mice (Figure 4(c)). In addition, immunohistochemical staining showed a lower proportion of PD-L1 positive cells in tumors from mice treated with cilengitide (Figure 4(d)). These results suggested that cilengitide downregulated PD-L1 expression in melanoma tissues in vivo.

### Cilengitide enhances anti-PD1 efficacy in vivo

**Figure 5.**
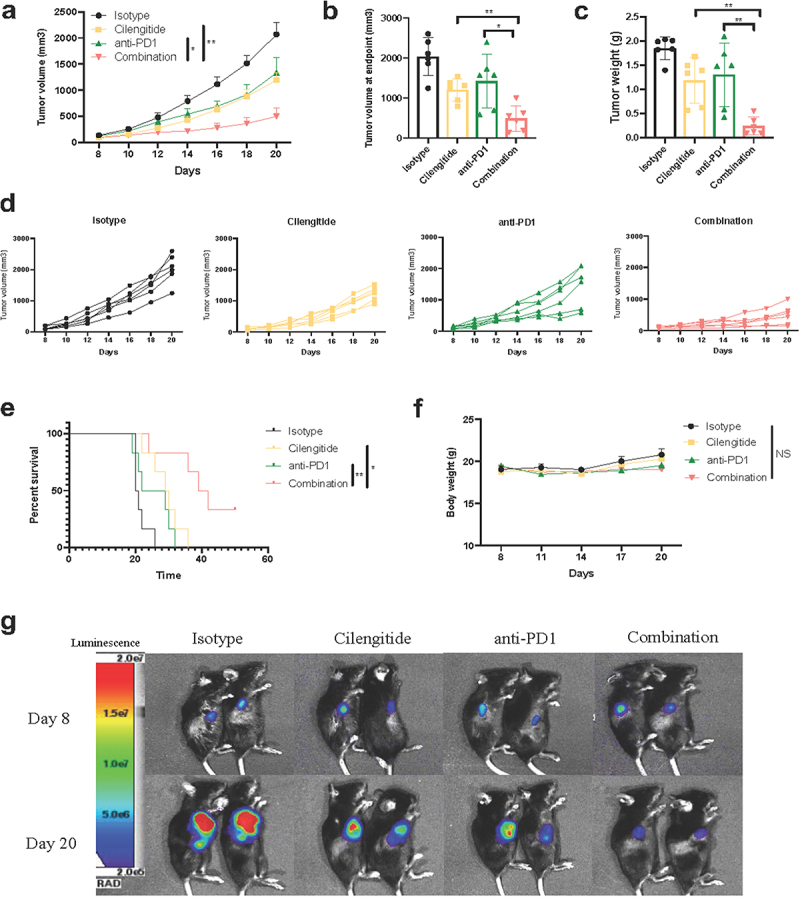
**Cilengitide enhances the in vivo antitumor effect of anti-PD1 antibody in B16 murine melanoma model**. (A, B, and D) Tumor volumes of C57BL/6 mice in different treatment groups (n = 6). (c) Quantified results of tumor weights at day 21 (n = 6). (e) Survival time of mice in different treatment groups (n = 6). (f) Quantified results of mice’s body weight (n = 6). (g) Tumors were detected by bioluminescence assay at 8 and 20 days after tumor implantation (n = 2). Data were represented as mean ± standard deviation. *p < 0.05, **p < 0.01, ***p < 0.001, NS, not significance (Student’s t test) and *p < 0.0332, **p < 0.0021, ***p < 0.0002, NS, not significance (Kaplan-Meier survival analysis). The in vivo experiments were repeated three times.

We evaluated the antitumor effect of cilengitide alone or in combination with anti-PD1 in a murine B16 melanoma model. Mice were randomly divided into four groups (n = 6): mice treated with cilengitide or anti-PD1 alone, the combination of cilengitide and anti-PD1, or PBS and isotype control. Treatments began at 8 days after subcutaneous implantation, when tumors became visible (Supplementary Fig. 2). At day 14, cilengitide showed moderate antitumor activity compared with the isotype control. Anti-PD1 antibody had an antitumor effect on some mice, however, other mice did not respond to anti-PD1 therapy. Tumor size in the anti-PD1 therapy group was smaller but not statistically different from the control mice. From day 16 onwards, the tumor volume in mice treated with cilengitide and anti-PD1 antibody was significantly smaller than in those that received monotherapy (Figure 5(a, b,&d)). At day 20, the average size of tumors in the control group reached 1933.35 mm3. According to the guidelines and regulations of the ethics committee of Tongji Medical College, day 20 was the latest time point at which tumor volume could be recorded before the mice were killed. At this point, the tumor weight in the combined treatment group was markably reduced than that in the other three groups (Figure 5(c)). Besides, survival was prolonged in mice that received combined cilengitide and anti-PD1 treatment. All mice who recieved the isotype control or monotherapy reached the observation end point at day 36 post-implantation, while the survival time of mice in the combination therapy group was extended to about 50 days (Figure 5(e)). On day 50 after treatment, the tumor volume of two mice in the combined treatment group was still less than 1000 mm^3^. Several basic and clinical studies have reported severe side effects associated with immunotherapy in combination with other treatments [32,33]. We therefore recorded the body weight of mice throughout the experiment. There was no significant difference in body weight between the four groups (figure 5(f)). To further assess differences in tumor volume between the four groups, we repeated our experiments using B16-luc cells and implemented bioluminescent imaging of B16-luc tumors on days 8 and day 20. The tumor center signal strength in the combined treatment group was significantly weaker than that in the other three groups at day 20 (Figure 5g).

### Combined cilengitide and anti-PD1 therapy promotes intratumoral T cell infiltration and function

**Figure 6.**
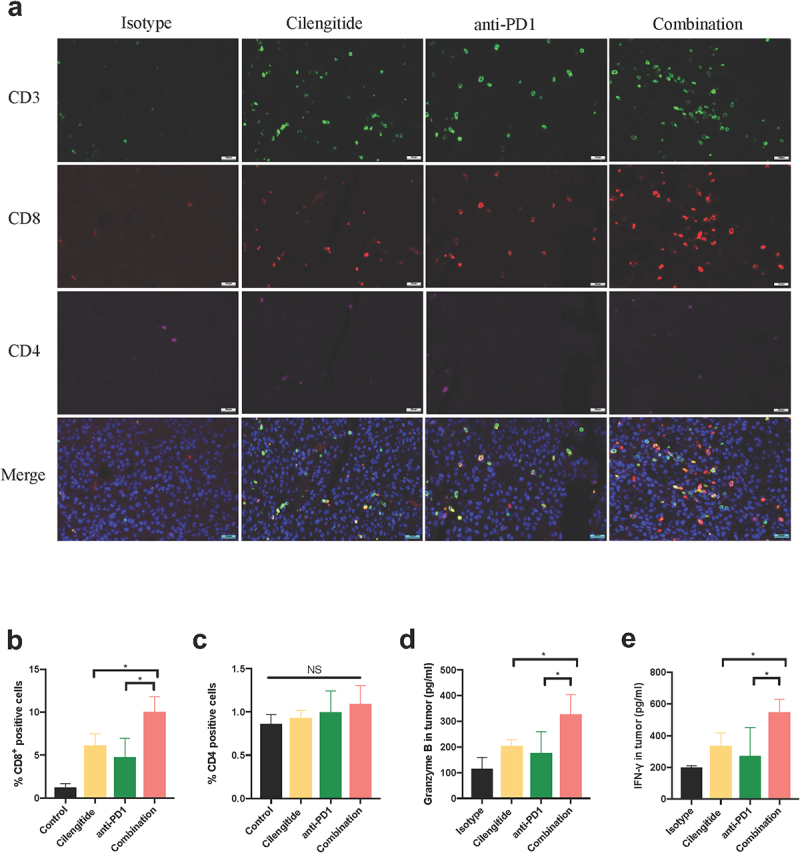
**Cilengitide potentiates anti-PD1 antibody efficacy by activating CD8^+^ T cell response**. (a) Representative images of immunofluorescence staining of CD3, CD4 and CD8 in tumors (n = 3). Scale bar: 100 µm. (b and c) Quantified results of CD4^+^ and CD8^+^ cells in tumors. (d) The contents of granzyme B in tumors determined by ELISA (n = 4). (e) The contents of IFN-γ in tumors determined by ELISA (n = 4). Data were represented as mean ± standard deviation. *p < 0.05, **p < 0.01, ***p < 0.001, NS, not significance (Student’s t test).

**Figure 7.**
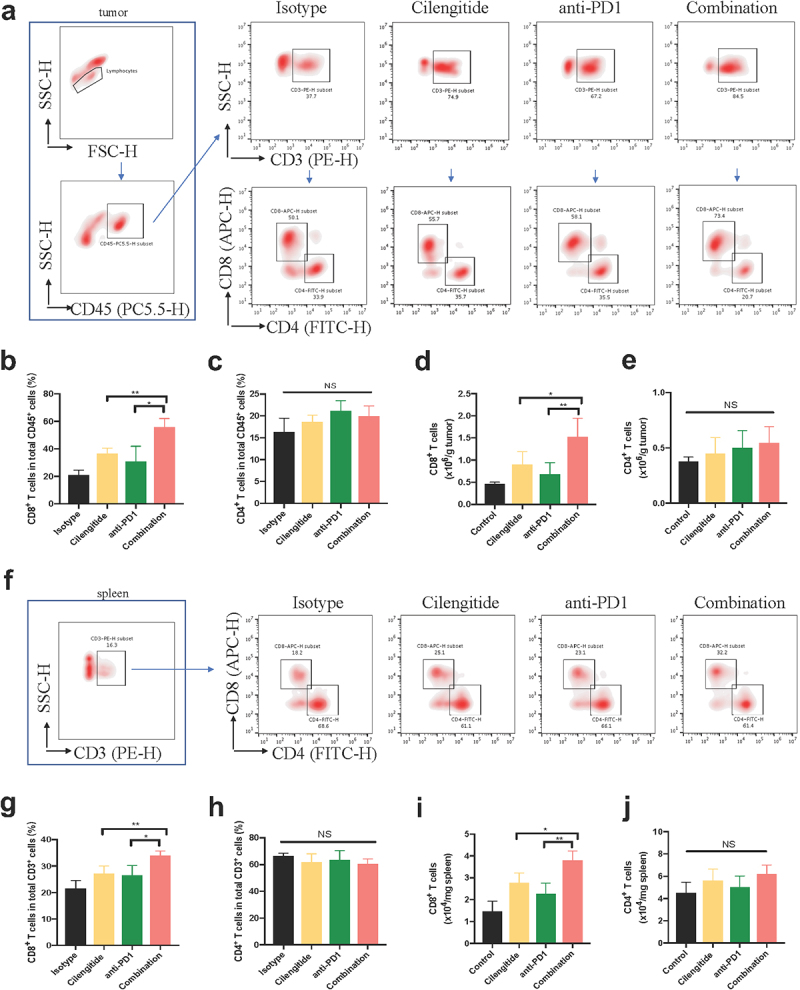
**Representative dot plots of CD8^+^ and CD4^+^ T cells in tumor and spleen tissues**. (a-c) The proportions of CD8^+^ and CD4^+^ T cells in total CD45^+^ cells in tumors detected by flow cytometry (n = 4). (d and e) The number of CD8^+^ and CD4^+^ T cells in tumors (n = 4). (f-h) The proportions of CD8^+^ and CD4^+^ T cells in total CD3^+^ cells in spleens of mice detected by flow cytometry (n = 4). (i and j) The number of CD8^+^ and CD4^+^ T cells in spleens. Data were represented as mean ± standard deviation. *p < 0.05, **p < 0.01, ***p < 0.001, NS, not significance (Student’s t test).

Most non-immunogenic tumors are characterized by a lack of TILs and/or TILs dysfunction. To determine whether cilengitide further enhances anti-PD1 therapy-mediated T cell cytotoxic activity in a murine melanoma model, we collected tumor and spleen tissues to analyze T cell infiltration and function at 21 days after treatment. The number of CD3^+^ and CD8^+^ TILs was considerably higher in the combined therapy group compared with the monotherapy group (Figure 6(a-c)). To measure IFN-γ and granzyme B levels in vivo, mice were euthanized at the endpoint, and tumors were fixed for ELISA detection. The concentrations of IFN-γ and granzyme B were 327.06 ± 76.14 pg/ml and 547.47 ± 81.31 pg/ml in the combined therapy group. Cilengitide combined with anti-PD1 antibody enhanced the release of IFN-γ and granzyme B compared with the isotype and monotherapy groups (Figure 6(d&e)). To further determine the ratio of CD3^+^CD8^+^ and CD3^+^CD4^+^ TILs in tumor and spleen tissues, flow cytometry was performed. The largest increase in CD3^+^CD8^+^ TILs infiltration was observed in the combined treatment group. The proportion of CD3^+^CD8^+^ TILs in CD45^+^ cells and the number of CD3^+^CD8^+^ TILs in tumors increased significantly in the combined treatment group. Nevertheless, there were no statistically significant differences in tumor CD3^+^CD4^+^ TILs infiltration among the four groups (Figure 7(a-e)). In addition, combined therapy also increased the proportion of CD8^+^ T cells in CD3^+^ cells and the number of CD8^+^ T cells in the spleens of mice. The infiltration of CD4^+^ T cells in spleens was not significantly different with monotherapy or combined therapy compared with the control (figure 7(f-j)).

## Discussion

Immune checkpoint inhibitors have been applied in clinical treatment for several solid tumors, and represent a significant innovation in cancer immunotherapy [34]. In recent years, many studies investigating combinations of therapies have been conducted to optimize immune checkpoint inhibitor therapy [35,36].

Integrins are abundantly expressed in tumor cells and tumor stromal cells, and are involved in cancer development, invasion, and metastasis. Our analysis of data from the Oncomine and TIMER online database revealed that the expression level of β3-integrin was significantly increased in SKCM and metastatic SKCM. Several studies have reported the link between increased expression of αvβ3-integrin and tumor growth, metastasis, and resistance to radiation therapy in melanoma [37–39]. In view of its abundant expression, αvβ3-integrin may be a promising therapeutic target in melanoma.

Cilengitide inhibits the proliferation and invasion of tumors expressing αvβ3-integrin in vitro and in vivo [40]. In our study, we observed reduced cell viability and enhanced apoptosis in melanoma cells with cilengitide treatment. Furthermore, our study showed the pathways involved in the tumor-inhibiting effect of cilengitide by transcriptome sequencing. Cilengitide alerted PI3K-AKT and MAPK signaling pathways in B16 cells, thereby affecting cell viability and apoptosis.

Our results revealed that cilengitide negatively regulated PD-L1 expression in murine and human melanoma cell lines in vitro by decreasing STAT3 phosphorylation. This regulatory effect was also observed in a murine melanoma model in vivo. Since the failure of the phase III clinical trial of cilengitide in glioblastoma, the application of cilengitide in other diseases has been reported. Japon and colleagues recently reported that cilengitide increased the sensitivity of breast cancer to paclitaxel and inhibited epithelial-to-mesenchymal transition (EMT) among paclitaxel-resistant cells [41]. Dong and colleagues reported that cilengitide reversed erlotinib resistance by inhibiting the Galectin-3/KRAS/RALB/TBK1/NF-κB signaling pathway in non-small cell lung cancer [42]. Our study revealed, for the first time, that cilengitide suppressed PD-L1 expression and STAT3 phosphorylation in melanoma, indicating a new therapeutic application for cilengitide.

To investigate the effect of combined therapy on the TME, we developed a murine melanoma model, in which mice were treated with cilengitide and anti-PD1 monoclonal antibody. Our results demonstrated that combining cilengitide with anti-PD1 monoclonal antibody enhanced CD8^+^ TILs infiltration in tumor and spleen tissues, thereby inhibiting local tumor growth and prolonging the survival period. The combination treatment had no significant effect on CD4^+^ TILs infiltration. Meanwhile, the combination treatment promoted cytotoxic T cell activity and generated a synergistic antitumor effect. Understanding the role of αvβ3-integrin in T cell activation may inform optimal combination immunotherapeutic strategies for patients.

There are some limitations to our study. Only one type of murine melanoma cell was available in CCTCC, so the combined antitumor effect of cilengitide and anti-PD1 monoclonal antibody was only verified in this model. Besides, our experiments only analyzed the changes in CD4^+^ and CD8^+^ TILs infiltration in tumor and spleen tissues, and the effect of the different treatments on other immune cells was not explored. Finally, the mechanism of the immune activating effect of cilengitide combined with anti-PD1 monoclonal antibody requires further exploration.

Taken together, this study focused on the potential role of αvβ3-integrin inhibitor in immunotherapeutic strategies for melanoma. Our research established a novel treatment option targeting αvβ3-integrin to activate the antitumor response by reusing available antitumor drugs.

## Supplementary Material

Supplemental Material

## Data Availability

Data used to support the findings of this study are included within the article.
