# Supplementary material for: Cilengitide, an αvβ3-integrin inhibitor, enhances the efficacy of anti-programmed cell death-1 therapy in a murine melanoma model
Source: Bioengineered. 2022 Feb 10;13(2):4557–72. doi: 10.1080/21655979.2022.2029236 (PMC8974133; doi:10.1080/21655979.2022.2029236)
Supplement: Supplemental Material [file KBIE_A_2029236_SM2770.zip › supplementary/Supplementary materials (1).docx]

**Supplementary Figure 1. Certificate of cell purchase.**


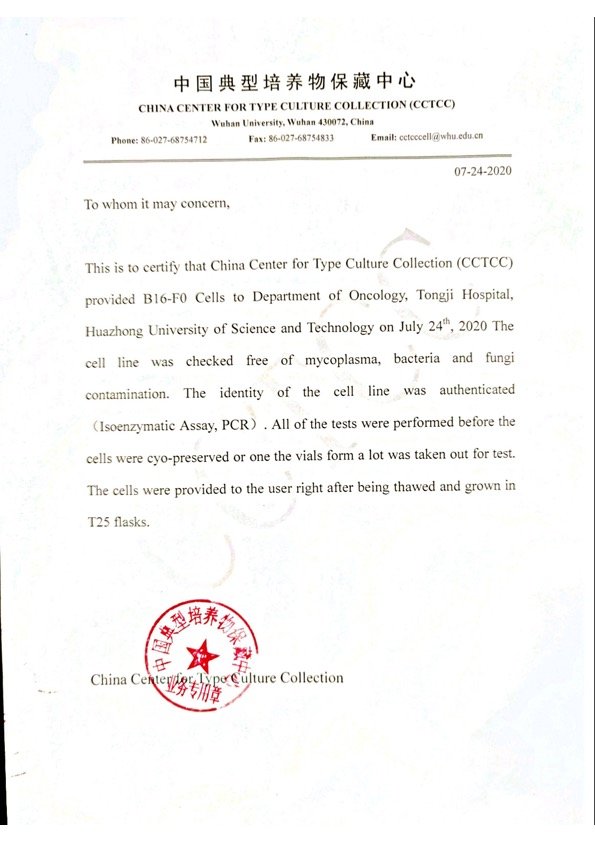


**Supplementary Figure 2. Methods of in vivo studies.**

**Supplementary Figure 3. Unedited gel images with markers of western blot.**


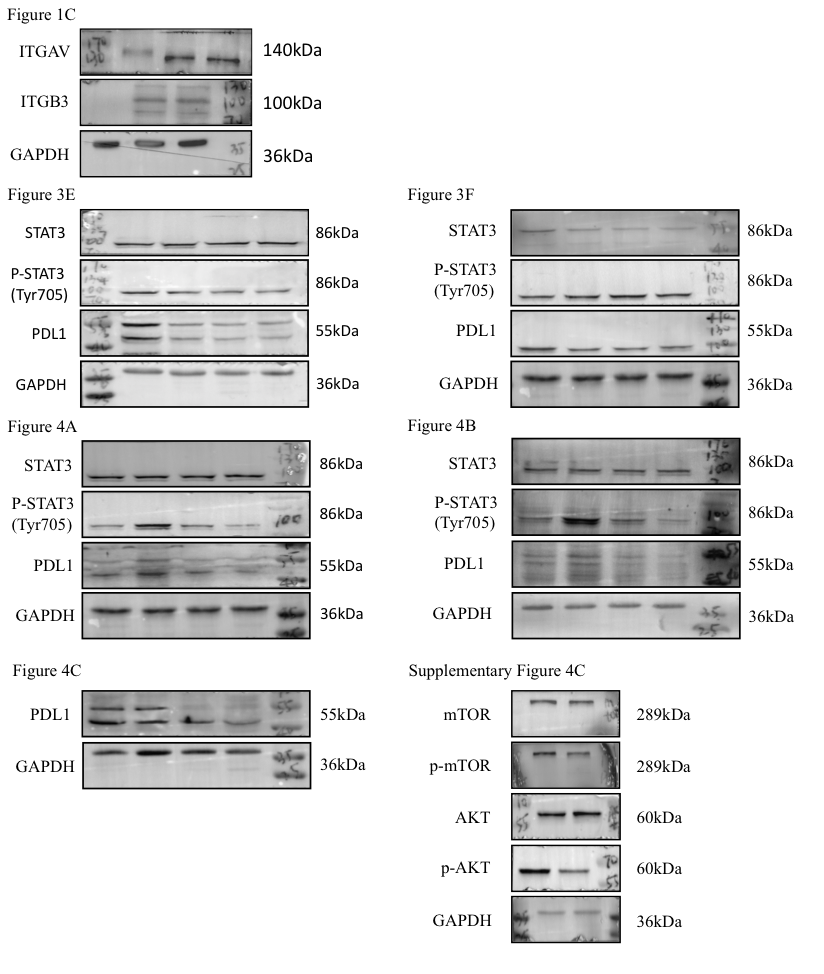


**Supplementary Figure 4. The protein expression of β3-integrin in HPA and the changed pathways after treatment with 5 µg/ml cilengitide.** (A) Protein expression of β3-integrin in normal skin tissue and melanoma tissue in HPA database. (B) The expression of the PI3K-AKT and MAPK signaling pathway related genes in normal B16 cells and cilengitide treated B16 cells detected by RT-qPCR. (C) The phosphorylation of AKT and mTOR in normal B16 cells and cilengitide treated B16 cells detected by western blotting. Data were represented as mean ± standard deviation. *p<0.05, **p<0.01, ***p<0.001, NS, not significance (Student’s t test). Each experiment was repeated three times.

**Supplementary Table 1. Primary antibodies used in western blot**

| Antibody | Catalogue | Dilution | Host |
| --- | --- | --- | --- |
| ITGAV | Abclonal A19017 | 1:1000 | Rabbit |
| ITGB3 | Abclonal A19037 | 1:1000 | Rabbit |
| PD-L1/CD274 | Proteintech 17952-1-AP | 1:1000 | Rabbit |
| STAT3 | CST 9139 | 1:1000 | Mouse |
| p-STAT3 (Tyr705) | CST 9145 | 1:1000 | Rabbit |
| mTOR | CST 2971 | 1:1000 | Rabbit |
| p-mTOR (Ser2448) | CST 5536 | 1:1000 | Rabbit |
| AKT | CST 9272 | 1:1000 | Rabbit |
| p-AKT (Ser473) | CST 4060 | 1:1000 | Rabbit |
| GAPDH | CST 5174 | 1:1000 | Rabbit |

**Supplementary Table 2. Antibodies used in flow cytometry**

| Antibody | Catalogue | Dilution | Host |
| --- | --- | --- | --- |
| APC anti-mouse CD274 antibody | Biolegend 124311 | 1:100 | 10F.9G2 |
| PE anti-human CD274 antibody | Biolegend 329705 | 1:100 | 29E.2A3 |
| APC rat IgG2b, κ isotype antibody | Biolegend 400611 | 1:100 | RTK4530 |
| PE mouse IgG2b, κ isotype antibody | Biolegend 400311 | 1:100 | MPC-11 |
| PerCP/cy 5.5 anti-mouse CD45 antibody | Biolegend 103131 | 1:100 | 30-F11 |
| PE anti-mouse CD3ε antibody | Biolegend 100308 | 1:100 | 145-2C11 |
| APC anti-mouse CD8α antibody | Biolegend 100712 | 1:100 | 53-6.7 |
| FITC anti-mouse CD4 antibody | Biolegend 100405 | 1:100 | GK1.5 |

**Supplementary Table 3. Primer sequences used in RT-qPCR**

| Gene | Sequences |
| --- | --- |
| Dusp6-F | 5′-ATAGATACGCTCAGACCCGTG-3′ |
| Dusp6-R | 5′-ATCAGCAGAAGCCGTTCGTT-3′ |
| Dusp7-F | 5′-TCGTCGCACATTGAGACGG-3′ |
| Dusp7-R | 5′-CTTGTCGGCATGGTTGGGAAT-3′ |
| Nfkb1-F | 5′-ATGGCAGACGATGATCCCTAC-3′ |
| Nfkb1-R | 5′-CGGAATCGAAATCCCCTCTGTT-3′ |
| Irs1-F | 5′-CGATGGCTTCTCAGACGTG-3′ |
| Irs1-R | 5′-CAGCCCGCTTGTTGATGTTG-3′ |
| Myc-F | 5′-ATGCCCCTCAACGTGAACTTC-3′ |
| Myc-R | 5′-GTCGCAGATGAAATAGGGCTG-3′ |
| Rasgrp2-F | 5′-GCTCCGTGGTTGCATCGAA-3′ |
| Rasgrp2-R | 5′-AGGAAGGTATGTACCAGGGGT-3′ |
| GAPDH-F | 5′-GGAGCGAGATCCCTCCAAAAT-3′ |
| GAPDH-R | 5′-GGCTGTTGTCATACTTCTCATGG-3′ |
